# One stone, two birds: The barley NLR protein MLA3 recognizes the rice blast fungus effector Pwl2 in addition to its cognate effector AVR_a3_ from barley powdery mildew

**DOI:** 10.1093/plcell/koad275

**Published:** 2023-10-31

**Authors:** Leiyun Yang

**Affiliations:** Assistant Features Editor, The Plant Cell, American Society of Plant Biologists; Department of Plant Pathology, College of Plant Protection, Key Laboratory of Integrated Management of Crop Diseases and Pests, Ministry of Education, Nanjing Agricultural University, Nanjing 210095, China; The Key Laboratory of Plant Immunity, Nanjing Agricultural University, Nanjing 210095, China

Plant nucleotide-binding leucine-rich repeat (NLR) proteins are intracellular immune receptors that recognize effector proteins secreted by pathogens to initiate robust immune responses. The majority of characterized NLRs recognize specific effectors from a single pathogen species, conferring plant resistance with high specificity (Kourelis and van der Hoorn 2018). To broaden the pathogen recognition spectrum, a subset of NLRs surveil common host proteins targeted by effectors from multiple pathogens (Kim et al. 2023). Previous examples of this phenomenon have involved indirect effector recognition by distinct host NLRs. The recognition of multiple pathogens by a single NLR protein is rarely reported.

In new work, **Helen Brabham, Diana Gómez De La Cruz, and colleagues** (Brabham et al. 2023) show that the NLR protein MLA3 encoded by the barley *Mildew locus a* (*Mla*) locus recognizes multiple pathogens. This NLR protein recognizes two structurally distinct effector proteins: AVR_a3_ from the barley powdery mildew *Blumeria graminis* f. sp. *Hordei* (*Bgh*) and Pathogenicity toward Weeping Lovegrass2 (Pwl2) from the blast pathogen *Magnaporthe oryzae*.

The barley *Mla* locus is known to contain three NLR gene families: *RGH1/Mla3*, *RGH2*, and *RGH3*, with the ability to detect a wide array of pathogens including *Bgh* (Wei et al. 2002). The barley cultivar Baronesse also confers resistance to *M. oryzae* through the NLR gene *Reaction to Magnaporthe oryzae1* (*Rmo1*), which had been genetically mapped to the *Mla* locus, known as *Mla3* in Baronesse (Inukai et al. 2006). To pinpoint the causal gene within the *Mla3* locus, the authors conducted a high-resolution recombination screen and found a strong genetic linkage between *Rmo1* and *Mla3*. Combinatory genomic analyses, such as RenSeq-PacBio and chromosome sequencing, revealed that the *Mla3* locus contains all three NLR families: *RGH1*/*Mla3*, *RGH2*, and *RGH3*, with four copies of *Mla3* and one copy each of *RGH2* and *RGH3*. Transcriptome analysis from the first leaf of Baronesse revealed that *Mla3*, *Mla3Δ6* with a six-base pair deletion, *RGH2*, and *RGH3* were expressed, suggesting that these four genes were potential candidate genes for conferring *Rmo1* resistance.

**Figure 1.**
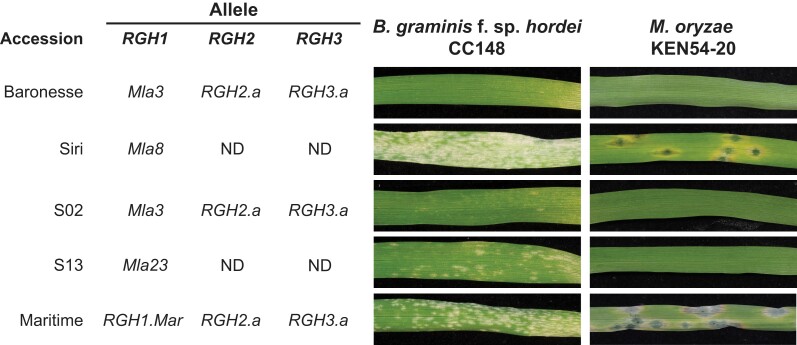
The disease resistance assay on the barley natural accessions carrying different alleles of *RGH1*, *RGH2*, and *RGH3*. Accessions carrying *RGH1/Mla3* showed full resistance to both *Bgh* and *M. oryzae*. ND, genes not detected in RNA-seq data. Reprinted from Brabham et al. (2023), Figure 4.

To identify the causal gene, the authors took advantage of the diverse natural variation within the *Mla* locus in barley. They discovered that the Maritime accession contained identical *RGH2* and *RGH3* but a different *Mla* compared to Baronesse. A disease resistance assay confirmed the expected resistance of Baronesse to the *M. oryzae* isolate KEN54-20, which contains the *AVR-Rmo1* effector. However, Maritime exhibited a susceptibility phenotype, indicating that *RGH2* and *RGH3* were not causal genes. They further found 11 near-isogenic lines in the Siri accession background that only differed in their *Mla* genes (Kølster and Stølen 1987). The disease resistance assay showed that line S02 containing *Mla3* and line S13, which carries *Mla23* with 98% sequence similarity at the DNA and protein levels to *Mla3*, exhibited resistance to the *M. oryzae* isolate KEN54-20 (see Fig. 1). This suggested *Mla3* was the causal gene for resistance. To validate this, the authors individually transformed all four candidate genes into a susceptible accession and assessed disease resistance in the transgenic T1 plants against the *Bgh* isolate CC148 (AVR_a3_) and the *M. oryzae* isolate KEN54-20. Only the *Mla3* transgenic plants exhibited resistance to both pathogens. Therefore, the gene responsible for resistance to *M. oryzae* is *Mla3*, which recognizes AVR_a3_ in *Bgh* and AVR-Rmo1 in *M. oryzae*.

To identify the effector *AVR-Rmo1*, the authors mutagenized spores of the *M. oryzae* isolate KEN54-20 using UV light and screened for gain-of-virulence mutants on Baronesse, and 12 such mutants were found. Whole-genome sequencing revealed that all of these mutants contained a deletion of the known effector gene *PWL2*. Structural analysis showed that Pwl2 belongs to the group of MAX (*Magnaporthe oryzae* Avrs and ToxB) effectors, which is structurally different from AVR_a3_, potentially containing RNAse-like folds. Transforming the *PWL2* gene into the mutants restored the resistance of Baronesse to the *M. oryzae* isolate KEN54-20, confirming that *PWL2* is *AVR-Rmo1*.

This led the authors to test whether the *Mla3* gene triggers cell death, a typical immune response in effector-triggered immunity, upon its recognition of Pwl2. As expected, coexpression of *MLA3* with *Pwl2* caused cell death in *Nicotiana benthamiana* leaves. The authors further explored whether this recognition involved an interaction between MLA3 and Pwl2 using a co-immunoprecipitation assay. A successful co-immunoprecipitation of MLA3 and Pwl2 was observed, marking the first report of an *MLA* allele co-immunoprecipitating with its cognate effector in planta.

In summary, this study unveils the molecular mechanism underlying the resistance of barley Baronesse to *M. oryzae*. The NLR MLA3 from Baronesse directly recognizes the Pwl2 effector from *M. oryzae*, resolving the long-standing question of Baronesse's resistance to *M. oryzae*. It also showcases the capability of a single NLR protein to recognize structurally distinct effector proteins from taxonomically different pathogens. Future work is needed to unravel the mechanisms by which MLA3 recognizes AVR_a3_ and Pwl2. The knowledge gained from this study advances our understanding of plant-microbe interactions and has practical implications for crop improvement.
